# Fermented rice polishings dietary supplementation sustainably enhanced growth performance, gut morphology, immune response and antioxidant status of Nile tilapia

**DOI:** 10.1038/s41598-025-26417-y

**Published:** 2025-11-21

**Authors:** Amr Ismail Zaineldin, Mohammed Barakat, Mohammed S. El Gohary, Mohammed Rashed, Ehab Elsebaey

**Affiliations:** 1 Aquatic Animal Nutrition, Aquaculture Department, Faculty of Aquatic and Fisheries Sciences, Kafrelsheikh University, Kafrelsheikh, 33516 Egypt; 2 Unit of Biochemistry, Nutritional Deficiency Diseases and Toxicology, Agriculture Research Center (ARC), Animal Health Research Institute (AHRI), Kaferelsheikh Branch, Giza, 85871 Egypt; 3 Unit of Fish Diseases, Agriculture Research Center (ARC), Animal Health Research Institute (AHRI), Kaferelsheikh Branch, Giza, 85871 Egypt; 4 Unit of Food Hygiene, Agriculture Research Center (ARC), Animal Health Research Institute (AHRI), Kaferelsheikh Branch, Giza, 85871 Egypt

**Keywords:** Fermented rice polishings, Growth, Gut morphology, Immunity, Antioxidant activity, Nile tilapia, Biotechnology, Immunology, Plant sciences

## Abstract

Fermentation of plant-derived by-products, including rice polishings (RP), is suggested for the sustainability of the aquafeed industry. In this study, fermented rice polishings (FRP) were incorporated into Nile tilapia diets at various levels: 0, 100, 200, 300, and 400 g/kg. Nile tilapia with an average initial weight of 19.25 ± 0.25 g/fish were divided into five groups (triplicates) and fed five test diets (R0, R10, R20, R30, and R40) for 85 days. Dietary FRP substantially increased final body weight, weight gain, specific growth rate, and protein efficiency ratio, but decreased feed conversion ratio compared to the control group (R0). According to regression analysis, dietary FRP at 210 to 230 g/kg leads to optimal growth performance. Fish fed with higher levels of FRP (R20, R30, and R40) showed greater crude lipid content in the carcass compared to R0 and R10. Graded FRP levels considerably enhanced intestinal architexture, particularly at R20 and R30 levels. Fish fed FRP showed a dose-dependent increase in digestive enzyme activity up to 300 g/kg (R30), with the R20 having the greatest activity. Serum lysozyme levels increased significantly across all FRP levels. Serum immunoglobulin activity enhanced considerably in the R20 and R30 groups. R20 had the greatest lysozyme and immunoglobulin activity. Fish given graduated FRP levels had higher levels of total antioxidant capacity, SOD, and CAT compared to R0, with R20 exhibiting the greatest activity. Ultimately, dietary FRP can be added at 210–230 g/kg to enhance the growth performance, productivity, gut shape, digestive enzyme activity, blood health, immunity, and antioxidant response of Nile tilapia.

**Supplementary Information:**

The online version contains supplementary material available at 10.1038/s41598-025-26417-y.

## Introduction

The global aquaculture sector has expanded to enhance food security and livelihoods, particularly in emerging economies where there is a growing demand for cost-effective and sustainable fish production^1^. Nile tilapia can withstand rigorous farming conditions, intensification, and rather poor water quality^2^. Nonetheless, Nile tilapia requires a specific number and quality of nutrients in order to achieve optimal growth performance, health, and production. Traditionally, fishmeal and soybean meal have served as the primary protein sources in Nile tilapia diets. However, their high prices, limited availability, and competition for human consumption restrict their use in aquafeed production^3^. Aquafeed contributes for 60–70% of the entire production costs in Nile tilapia farming^4,5^; hence, sustainable alternative feed ingredients are required. Integrating plant products rich in protein, fat, and minerals is crucial for the aquaculture feed industry^6^. The incorporation of plant by-products into feed formulations is constrained by limited availability of plant-based ingredients and their high costs^7,8^. The ongoing global conflicts, which have led to soaring prices of feed ingredients and additives, further emphasize the importance of exploring non-traditional feed materials for the sustainable aquaculture feed industry^8^. Among the many parts of rice grains are bran, hulls, and rice polishings, which are frequently utilized in animal feed. Rice polishing, a process that involves the removal of the outer layers of rice grains to enhance their appearance and shelf life, generates a significant byproduct known as rice polishings^9,10^. More precisely, rice polishings (RP) are the main by-product of brown rice milling, which consists of peel, aleurone layer, hypo aleurone layer, seed coat, nucellar layer, a small number of plumule and broken rice^11^. It is considered as a low-cost by-product with well-known health benefits, including high fiber (20%), high protein (14%) and fat (14.75%), as well as other carbohydrates, vitamins, minerals, and essential unsaturated fatty acids^12^. It also contains antioxidants like phenolics, tocopherols, tocotrienols, and γ-oryzanol^13^. Additionally, RP is becoming increasingly and more popular in the feed, pharmaceutical, and nutraceutical sectors because of its high nutritional content, affordability, accessibility, and potential for high bioactivity, as well as the related health advantages^14^. But because of a number of challenges with the processing, including raw ingredients that are prone to rancidity and poor functioning, it is still mostly utilized as feed^15,16^. Consequently, there is a growing research focus in cereal science on utilizing rice bran as a primary raw material for developing diverse nutritious feeds, improving the processing characteristics of rice polishings, and addressing the challenge of maintaining the freshness of rice polishings through modern feed processing technologies^15^. Here, it examines and forecasts rice polishings stabilization, fermentation, biological enzymolysis, the use of all rice polishings constituents in feed, and rice polishings processing in order to offer a guide for rice polishings processing and use in Nile tilapia feeding.

## Materials and methods

### Solid state fermentation of rice Polishings (SSF-RP)

The commercial rice polishing powder was fermented with baker’s yeast (*S. cerevisiae*; viable cell counts 1.5 × 10^10^ cell g^− 1^ SIGMA, Baker’s Yeast Type II) using the technique described by ^17^. In a 5 L glass Jar, 2 kg of RP + 66.6 mg dry yeast + 100 mL molasses + 1.6 L distilled water were added and then mixed for 15 min. The mixture was kept and the glass jar covered with aluminum foil at 30 °C for 72 h. After fermentation was completed, conventional sun drying was performed. 10 g of FRP was tested after 72 h of fermentation to determine the phytic acid according to ^18^ methedology before and after fermentation. Additionally, the chemical composition of RP before and after fermentation was estimated according to the^19^ methods.

### Diets formulation

The formulation of five experimental diets that are isonitrogenous (28.5% crude protein) and isocaloric (19.07 MJ kg^-1^ gross energy) is fulfil the nutritional requirements of Nile tilapia according to NRC^20^ (Table 1). The first diet is the control diet (0%, free of FRP). The inclusion levels of FRP were 10%, 20% ,30 and 40% in the other four experimental diets. All dry ingredients were finely ground, accurately weighed, and manually premixed for 5 min. The mixture was then transferred to a mechanical food mixer and blended for an additional 15 min to ensure homogeneity. Soybean oil was gradually incorporated during this stage to achieve uniform distribution. The homogenized mixture was subsequently pelleted using a laboratory-scale pellet mill (California Pellet Mill, San Francisco, CA, USA) fitted with a 1.6–2.1 mm diameter die. The resulting pellets were air-dried at ambient temperature (25 ± 2 °C) for 24 h to reduce moisture content. Finally, the dried pellets were packed in airtight laboratory-grade containers and stored at − 20 °C until use to preserve nutritional integrity.

**Table 1:** Dietary formulation and proximate composition of the experimental diets.

Ingredients (g kg^−1^ dry diet)	Test diets				
	R0	R1	R2	R3	R4
Yellow corn	488	418	343	263	193
Rice polishings	0	100	200	300	400
Soya bean meal 46%	360	350	330	310	280
Fish meal	55	50	50	50	50
Glutin^1^	60	50	50	50	50
Mono-calcium phosphate	6	6	6	6	6
Fish oil	15	10	5	5	5
Calcium carbonate	5	5	5	5	5
NaCl	5	5	5	5	5
Sodium bicarbonate	1	1	1	1	1
Stay C^2^	0.8	0.8	0.8	0.8	0.8
Vitamins mixture^3^	1	1	1	1	1
Minerals mixture^4^	1	1	1	1	1
Choline chloride	1	1	1	1	1
Anti- mycotoxin	1	1	1	1	1
Proximate analysis%					
Dry matter	90.6	90.7	90.6	90.5	90.2
Crude protein	27.4	27.3	27.44	27.65	27.5
Crude lipid	4.72	5.13	5.63	6.62	7.62
Crude fiber	3.48	4.59	5.64	6.68	7.68
Crude ash	4.1	4.4	4.8	5.1	5.4
Energy (kJ g^−1^)**	19.29	19.32	19.4	19.65	19.77
Amino acid analysis %					
Lysine	2.6178	2.8021	2.9856	3.1556	3.3256
Methionine	1.2502	1.2935	1.33985	1.37745	1.42635
Arginine	3.61325	3.943	4.2639	4.5623	4.8749
Histidine	1.8473	1.8693	1.8909	1.8995	1.9224
Isoleucine	2.5736	2.8109	3.0556	3.2838	3.5261
Leucine	2.5124	2.9836	3.5294	4.0692	4.5889
Phenylalanine	3.2211	3.272	3.3365	3.3785	3.4465
Threonine	2.603	2.6648	2.7328	2.7828	2.8528
Valine	3.438	3.5689	3.7069	3.8209	3.9619

The experimental diets design: R0: Control unreplaced diet (0% Rice polishings supplementation level), R10: 10% Rice polishings supplementation level, R20: 20% Rice polishings supplementation level, R30: 30% Rice polishings supplementation level, R40: 40% Rice polishings supplementation level.

^1^Glutin: Corn gluten meal (62% crude protein).

^2^Stay-C 35.

^3^Vitamin mixture (g/kg diet): 0.10 β-carotene; 0.01 vitamin (D3); 0.05 vitamin (K3); 0.38 vitamin (E); 0.06 vitamin (B1); 0.19 vitamin (B2); 0.05 vitamin (B6); 0.0001 vitamin (B12); 0.01 biotin; 3.85 inositol; 0.77 niacine (nicotic acid); 0.27 Ca panthothenate ; 0.01 folic acid ; 7.87 choline choloride ; 0.38 ρ-aminobenzoic acid ; 1.92 cellulose.

^4^Mineral mixture (g/kg diet): 5.07 MgSO_4_; 3.23 Na_2_HPO_4_; 8.87 K_2_HPO_4_; 1.10 Fe citrate; 12.09 Ca lactate; 0.01 Al (OH)_3_ (Aluminum hydroxide); 0.13 ZnSO_4_; 0.004 CuSO_4_; 0.03 MnSO_4_; 0.01 Ca (IO_3_)^2^; 0.04 CoSO_4_.

**Calculated using combustion values for protein, lipid and carbohydrate of 236, 395 and 172 kJ g^− 1^, respectively and carbohydrate was calculated by the difference: 100- (protein + lipid + ash).

### Fish experiment

Healthy mono-sex Nile tilapia, *Oreochromis niloticus* were purchased from a private farm, Tolmbat 7, Kafr El-Sheikh Government, Egypt. Throughout the acclimation period (two weeks), fish were fed the control diet (3% of total biomass), which provided equal quantities at 9:00, and 1:00 p.m. at the aquaculture lab of Animal Health Research Institute, Kafr El-Sheikh, Egypt. The feed was estimated at 28.5% crude protein and 5.7% crude lipid. Following acclimation, 300 healthy Nile tilapia were split into five groups, each with three replicates, and with a stocking density of 20 fingerling per glass tank (60 L capacity). The average weight of the fish was 19.25 ± 0.25 g. Dechlorinated tap water was used to fill the tanks and compressed air provided for oxygen requirements compressed air provided for oxygen requirements. Every day, around one-third of the water volume in each tank was renewed after the accumulation of excreta was cleaned and removed. Fish were fed twice daily (at 9:00 a.m. and 1:00 p.m.) for 85 days. Throughout the trial, fish were weighed regularly to adjust feed quantities based on biomass in each tank.

Throughout the feeding trial, the water quality parameters that were measured were: temperature (23 ± 1.5 °C), pH (7.5 ± 0.7), dissolved oxygen (5.6 ± 0.3 mg/L), and ammonia (0.010–0.016 mg/L). These are considered to be ideal ranges for Nile Tilapia.

### Growth and feed utilization measures

Fish were collected at the end of the feeding trial, counted, measured the length, and weighed. Growth performance and feed utilization metrics were determined using standard Reference formulae according to NRC^20,21^. The following equations were used to calculate the growth indices:

- Weight gain (WG, %) = [(FBW – IBW) ×100] / IBW.
- SGR: specific growth rate (%/day) = 100 × [ln final body weight (g) − ln initial body weight (g)]/duration of feeding (day).
- FCR: feed conversion ratio = FI / WG.
- FCE: feed conversion efficiency = live weight gain (g)/dry feed intake (g).
- PER: protein efficiency ratio= [WG (g) / PI (g)] × 100.
- Survival (%) = 100 × (final number of fish/initial number of fish).

Randomly, five fish per tank were chosen and stored at − 20℃ until carcass proximate analysis was conducted.

### Diet and carcass composition

For both diets and fish samples, a standard technique for determining the nutritional content of the test diets and the chemical content of the whole fish body was applied in triplicates for diets and fish samples (AOAC, 2016). Crude protein (N factor = 6.25) was analyzed via the Kjeldahl apparatus (Labconco, Labconco, Kansas, MO, USA). Crude fat was extracted using the Soxhlet extraction method (Lab-Line Instruments, Melrose Park, IL, USA). For the ash, samples were burnt in the Muffle furnace (Thermolyne Corporation, Dubuque, Iowa, USA) at 550 °C for 6 h while the moisture content was determined using oven-drying at 110 °C to reach the constant weight. The system used for detecting the amine acids profile was high performance Amino Acid analyzer (Biochrom 30).

### Fish anesthesia and hematological analysis

After 24 h fasting, blood samples were obtained from the caudal vein of 5 fish anaesthetized with tricane methanesulfate (MS222 ; Sigma, St. Louis, MO, USA) (150 mg/L, buffered with sodium bicarbonate to neutralize pH). Anesthesia depth was confirmed by loss of equilibrium, cessation of opercular movement, and absence of response to a tail pinch stimulus. Once fully anesthetized, blood samples were collected via caudal vein puncture, then deposited into heparinized and non-heparinized Eppendorf centrifuge tubes, according to ^22^. plasma and serum were obtained After Eppendorf centrifugation at 5000 rpm/15 min. In Plasma, Total hemoglobin (Hb), red blood cells (RBCs) and white blood cells (WBCs) were determined using Semi- automatic analyzer for clinical chemistry and hematology tests- Model 2000 Evolution, EMEG using Bayer Diagnostics Reagents strips following the manufactory guidelines. Mean corpuscular volume (MCV), mean corpuscular hemoglobin (MCH), and mean corpuscular hemoglobin concentration (MCHC) were calculated using the method of Hawkey et al., ^23^.

The obtained serum was used for biochemical, immunological, and anti-oxidant analysis. Total cholesterol and triglyceride were assessed spectrophotometrically using an automated analyzer (SPOTCHEM™ EZ model SP-4430; ARKRAY, Inc., Kyoto, Japan) according to Tatsumi et al., ^24^. At a wavelength of 540 nm, calorimetric measurements were made of glucose, total protein levels, and the liver’s enzyme activity, such as alanine aminotransferase (ALT) and aspartate aminotransferase (AST)^25^.

Following the blood sampling, the viscera and liver were then dissected out from the fish, individually weighed, and measured (length) to calculate the condition factor, viscerosomatic index, and hepatosomatic index using the following formulas: 1-Condition factor = fish weight/ (fish length) (cm) × 100. 2-Viscerosomatic index = viscera weight/fish weight × 100. 3-Hepatosomatic index = liver weight/fish weight × 100.

### Gut morphology analysis

Fish were captured and anesthetized in a separate plastic container using clove oil (2–3 drops per liter of water) until fully sedated (2–3 min), following ARRIVE guidelines^26^. Once anesthetized, three fish per replicate tank were selected for intestinal morphology analysis. In accordance with standard ethical guidelines, cervical dislocation was performed to ensure death prior to dissection. The whole gastrointestinal tract were then aseptically collected, and the anterior, middle, and posterior parts of the intestine were taken, washed twice in twice in PBS (pH = 7.4), then were immersed in Davidson’s solution (agitated for 5 min) for 8 h. Following a progressive dehydration in ethanol (70%–100%), fixed tissues were twice cleaned with xylene (1 and 2 h)before being embedded in paraffin. 5-µm thickness Sections were stained with hematoxylin and eosin (H&E) and examined under light microscopy (Nikon, Tokyo, Japan) and a camera (Digital Sight DS2MV with a DS-L2 control unit; Nikon), with data analysed using SigmaScan Pro 5 software. Villi length, villi width, and goblet cell numbers were measured using Image J analysis software with magnifications of 100×, 200×, and 400×. For each tissue, ten measurements were obtained according to an established protocol^27^.

### Immunological assays

An Enzyme-Linked Immunosorbent Assay (ELISA) kit (Cusabio, Wuhan, Hubei, China) was used to measure the total immunoglobulin M (IgM) levels in the blood according to the manufacturer’s instructions. Lysozyme activity was evaluated according to the method described by Demers and Bayne^28^. The lysozyme unit present in serum (µg/mL) was obtained by comparison with a standard curve produced using lyophilized hen egg white lysozyme.

### Antioxidants analysis

Superoxide dismutase (SOD), and catalase (CAT) levels in serum were evaluated spectrophotometrically following the methods in previous studies^29,30^, respectively. Total antioxidants capacity (TAC) was analyzed in serum using colorimetric commercial kits purchased from Bio-Diagnostic Co. (Cairo, Egypt) according to Koracevic et al., ^31^.

### Digestive enzyme activity

Serum lipase activity was determined colorimetrically at the wavelength 580 nm using kits (Ref: 281 001; Spectrum, Egyptian Company for Biotechnology, Egypt)^32^. The serum amylase activity was determined colorimetrically at the wavelength 660 nm using commercial kits (Cat. No. AY1050, Biodiagnostic Co. Egypt)^33^. Also, serum protease activity was determined colorimetrically at the wavelength 450 nm following the manufacturer’s instructions (Number: 23263, Thermo Scientific Co., USA).

### Economical viability assessment

The economic analysis methodology followed established protocols^4,34^. The local market pricing for utilised ingredients were employed. These prices (US$) are as follows: herring fish meal, 1.25; soybean meal, 0.41; corn,0.25; rice polishings, 0.24; corn gluten, 0.97; mono calcium phosphate, 0.4; fish oil, 0.95; vitamin and mineral premix, 1.6.

At the end of the trial, the exchange rate was 1 USD = 50 Egyptian pounds (EGP). The following equations were used:

1. Cost reduction per ton gain (USD) = feed cost per kg gain of the control diet (R0) – feed cost per kg gain of R10,or R20,R30 or R40 (USD).
2. Cost reduction per kg gain (%) = 100 (cost reduction per kg gain [USD] in R10,or R20,R30 or R40 diets/feed cost per kg gain of the control diet [USD].

### Statistical analysis

Data were tested for normality (Shapiro–Wilk test) and homogeneity of variance (Levene’s test) at a significance level of 5%. Further, one-way analysis of variance (ANOVA) was performed, followed by Tukey–Kramer ’s multiple range test to compare mean values between treatments at (*P* < 0.05) using SPSS version 22 (SPSS Inc., IL, USA). All data are demonstrated as mean ± standard error (SE). Polynomial regression was employed to obtain the linear and quadratic effect of contributing graded level of fermented rice polishings on the response variables demonstrated.

### Ethical approval

The study protocol received ethical approval from the Animal Health Research Institute Ethics Committee (2024) in compliance with European Union Directive 2010/63/EU. All experimental procedures adhered to institutional guidelines and ARRIVE reporting standards (https://arriveguidelines.org). Research was conducted in controlled artificial pond facilities at the Animal Health Research Institute, Kafrelsheikh, Egypt, requiring no special access permissions. No human participants or protected species were involved in this study.

## Result

### Nutritional value and phytic acid content of rice Polishings after solid state fermentation

Table 2 shows the proximate analysis of RP and FRP; moisture, protein, fat, ash, and fiber. Protein content of RP was raised from 12.67 ± 0.2% to 17.25 ± 0.56% by yeast SSF. The lipid content in RP and FRP decreased from 13.72 ± 0.12% to 10 ± 0.5%, whereas the fibre content decreased from 19.2 ± 0.17% to 9.5 ± 0.23%. Additionally, Phytic acid concentration in raw RP was seen to decrease dramatically from 6.24 ± 0.09 mg g^-1^ to 1.23 ± 0.03 mg g^-1^ following SSF by *S. cerevisiae*.

**Table 2:** Rice Polishings proximate analysis before and after solid state fermentation.

Rice polishings	Nutrients%		
	Before SSF	After SSF	*P* value
Moisture (%)	8.6 ± 0.3	9.5 ± 03	0.23
Crude protein (%)	12.67 ± 0.2^b^	17.25 ± 0.56^a^	0.002
Crude lipid (%)	13.72 ± 0.12^a^	10 ± 0.5^b^	0.0001
Ash (%)	6.32 ± 0.1	6.15 + 0.04	0.44
Crude fiber (%)	19.2 ± 0.17^a^	9.5 ± 0.23^b^	0.0001
Phytic acid (mg g^−1^)	6.24 ± 0.09^a^	1.23 ± 0.03^b^	0.0001

Values are described as means ± SE. Different letters indicate significant differences (*P < 0.05*) while the absence of letters indicates no significant differences (*P>0.05*).

### Growth performance and feed efficiency

**Fig. 1:**
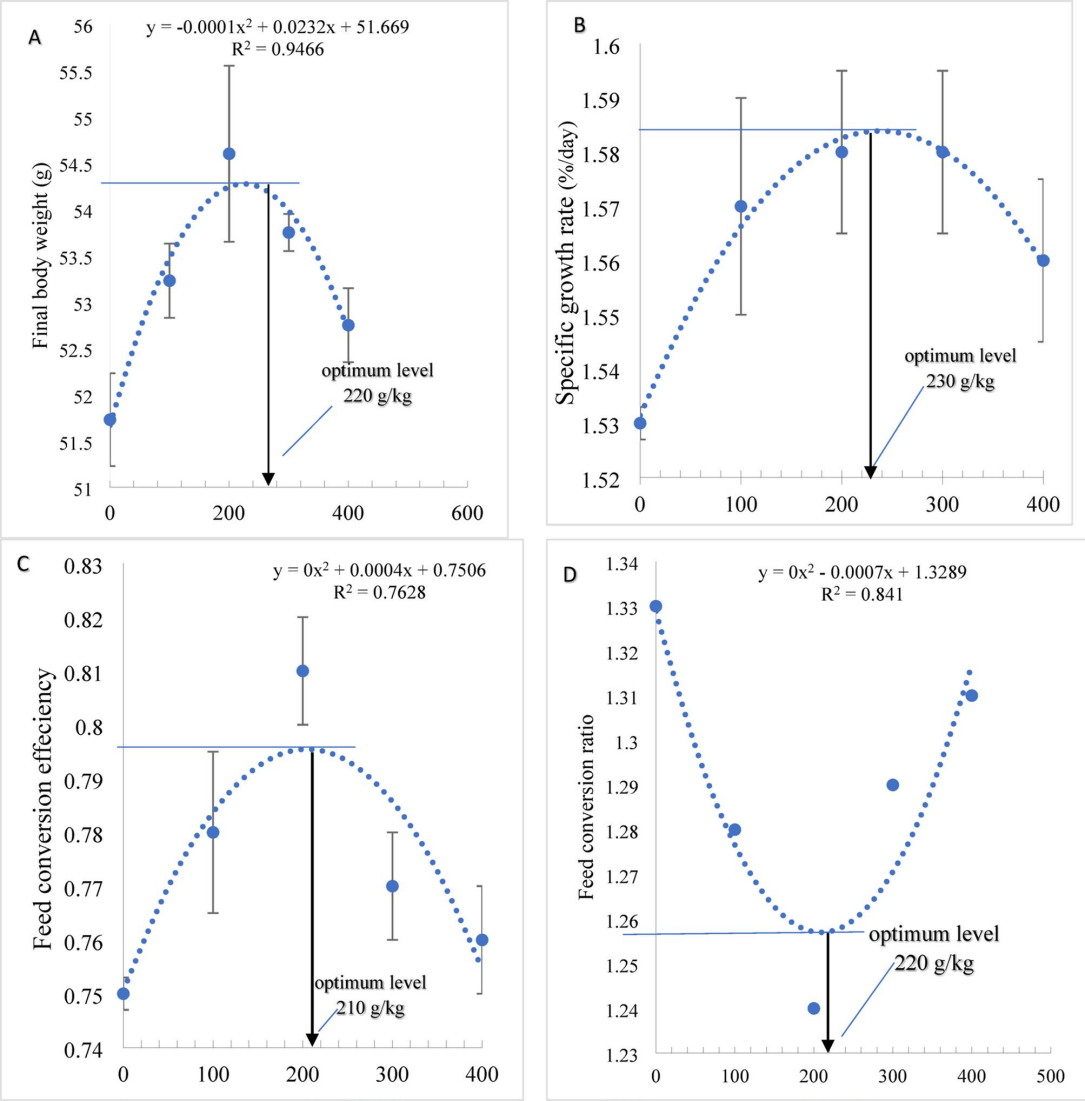
Polynomial regression analysis (*P* < 0.05) between final weight (**A**) and specific growth rate (**B**) ,feed conversion effeciency (**C**), and feed conversion effeciency (**D**) of Nile tilapia-fed dietary fermented rice hulls for 85 days. Values are described as means ± SE.

The results of the growth performance traits indicated that Nile tilapia fed R10, R20, R30, and R40 showed higher (*P* < 0.05) final body weight (FBW), weight gain (WG), specific growth rate (SGR), feed conversion efficiency (FCE), and protein efficiency ratio (PER) than fish fed R0 (Table 3). On the other hand, the feed conversion ratio (FCR) was markedly declined (*P* < 0.05) by dietary R10, R20, and R30 compared to fish-fed R40 and R0 (Table 3). The survival rate ranged between 91.5 and 95.3% without marked differences among the groups (*P*˃0.05). The regression analysis showed that the maximum FBW (Fig. 1A) SGR (Fig. 1B), FCE (Fig. 1C) and FCR (Fig. 1D) can be reached by dietary inclusion of fermented rice polishings (FRP) at 210 to 230 g/kg diet.

**Table 3:** Growth performance of nile tilapia, *Oreochromis niloticus* fed the experimental diets for 85 days.

Parameters	Test diets				
	R0	R10	R20	R30	R40
FBW	51.73 ± 0.5^b^	53.23 ± 0.4 ^ab^	54.6 ± 0.95 ^a^	53.75 ± 0.2^ab^	52.75 ± 0.4^ab^
WG	170.41 ± 1.01^b^	177.93 ± 2.17^ab^	180.01 ± 2.4^a^	178.53 ± 2.77^ab^	175.51 ± 0.9^ab^
SGR	1.53 ± 0.003b	1.57 ± 0.02^ab^	1.58 ± 0.015^a^	1.58 ± 0.015 ^ab^	1.56 ± 0.015 ^ab^
FCR	1.33 ± 0.03^a^	1.28 ± 0.03^ab^	1.24 ± 0.01^b^	1.29 ± 0.01^ab^	1.31 ± 0.01^a^
FCE	0.75 ± 0.003^b^	0.78 ± 0.025^ab^	0.81 ± 0.01^a^	0.77 ± 0.01^ab^	0.76 ± 0.01^b^
PER	2.6 ± 0.02 ^b^	2.75 ± 0.025^ab^	2.83 ± 0.01^a^	2.72 ± 0.025^ab^	2.68 ± 0.015^ab^
Survival(%)	92.5 ± 1.7	91.5 ± 1.85	91.7 ± 1.9	95.3 ± 0.85	91.5 ± 1.5

Values are described as means ± SE. Different letters indicate significant differences (*P < 0.05*) while the absence of letters indicates no significant differences (*P˃0.05*). FBW: final body weight (g); WG: Weight gain (WG, %) ; SGR: specific growth rate (%/day) ; FCR: feed conversion ratio; FCE: feed conversion efficiency; PER: protein efficiency ratio.

### Carcass composition

The body composition of Nile tilapia fed varying amounts of fermented rice polishings for 85 days is displayed in Table 4. There were no significant impacts on the moisture, crude protein, or ash contents (*P˃0.05*), while the crude lipid content was significantly impacted by the test diets (*P < 0.05*). Compared to fish fed other diets, fish fed dietary R20,R30,and R40 had a greater crude lipid content (*P < 0.05*).

**Table 4:** Whole body proximate analysis of nile tilapia, *Oreochromis niloticus* fed the experimental diets for 85 days.

Parameters	Test diets				
	R0	R10	R20	R30	R40
Moisture	68.7 ± 0.6	68.13 ± 1.1	68.35 ± 1.3	68.4 ± 1.6	68.5 ± 1.6
Crude protein	16.4 ± 0.5	16.6 ± 0.24	16.5 ± 0.3	16.4 ± 0.5	16.26 ± 0.8
Crude lipid	5.93 ± 0.4^c^	5.76 ± 0.5^c^	6.5 ± 0.2^b^	6.55 ± 0.6^b^	7.25 ± 0.6^a^
Crude ash	5.83 ± 0.3	6.03 ± 0.4	5.8 ± 0.3	6.1 ± 0.2	6.3 ± 0.5
CF (%)^1^	2.4 ± 0.02	2.57 ± 0.3	2.52 ± 0.15	2.55 ± 0.1	2.17 ± 0.04
HSI (%)^2^	1.78 ± 0.22	1.84 ± 0.24	1.83 ± 0.7	1.82 ± 1.2	1.69 ± 0.4
VSI (%)^3^	8.5 ± 0.5	8.45 ± 0.4	8.8 ± 0.34	8.5 ± 0.4	7.97 ± 0.1

Values are means of triplicate groups ± S.E.M. Within a row, means with the same letters are not significantly different (*P* > 0.05).^1^CF, condition factor ; ^2^HSI, hepatosomatic index; ^3^VSI, viscerasomatic index.

### Hematobiochemical indices

Significantly higher Hb, and RBC levels were observed in R10, R20 ,and R30 groups, with the highest values observed in the R20 group (*P < 0.05*) (Table 5). Furthermore, tilapia fed the R40 diet had similar Hb and PCV as fish fed the control diet (R0). Packed cell volume demonstrated significant high results in all fermented rice polishings supplied diets (R10,R20,R30,and R40) versus control one (R0)(*P < 0.05*). MCV, MCH, and MCHC demonstrated decreased levels with fermented rice polishings supplementation levels R10, R20, R30, in contrast, R40 and R0 demonstrated the higher levels. All diets supplemented with fermented rice polishings exhibited a considerable increase in heterophil populations (*P < 0.05*), with the exception of the highest level (R40), which had a lower level similar to that in the control group (R0).

The effects of the graded levels of FRP on the blood biochemical traits of Nile tilapia are shown in Table 6. No marked effects on the glucose, triglycerides, total cholesterol, and ALT(*P > 0.05*). R10, R20, and R30 revealed significantly higher levels of serum total protein and lower levels of AST as compared to the control (R0) and R40 groups (*P < 0.05*).

**Table 5:** Hematological parameters of nile tilapia, *Oreochromis niloticus* fed the experimental diets for 85 days.

Parameters	Test diets				
	R0	R10	R20	R30	R40
Hemoglobin (Hb, g dl−1)	4.17 ± 0.09^b^	4.5 ± 0.04^ab^	4.8 ± 0.05^a^	4.55 ± 0.15^ab^	4.25 ± 0.08^b^
Red blood cells (RBCs, ×106 µl−1)	2.01 ± 0.07^c^	2.34 ± 0.04^b^	2.7 ± 0.03^a^	2.38 ± 0.12^ab^	1.95 ± 0.06^c^
Packed cell volume (%)	19.5 ± 0.4^c^	21.29 ± 0.1^ab^	21.85 ± 0.16^a^	20.85 ± 0.3^ab^	20.5 ± 0.2^bc^
Mean corpuscular volume (MCV, fl.)	97.4 ± 3.9^ab^	91.02 ± 1.1^bc^	81.05 ± 0.3^c^	87.97 ± 3.6^bc^	105.5 ± 3.8^a^
Mean corpuscular hemoglobin (MCH, pg)	20.8 ± 0.9^ab^	19.3 ± 0.16^bc^	17.9 ± 0.03^c^	19.18 ± 0.6^bc^	21.8 ± 0.3^a^
Mean corpuscular hemoglobin concentration (MCHC, g dL-1)	21.4 ± 0.1^ab^	21.25 ± 0.1^ab^	22.1 ± 0.1^a^	21.8 ± 0.4^a^	20.7 ± 0.5^b^
White blood cells (WBCs, ×103 µl − 1)	14.5 ± 0.6	14.6 ± 0.3	15 ± 0.5	15.6 ± 0.25	14.4 ± 0.5
Heterophil (%)	26.6 ± 0.7^c^	32.12 ± 1.06^b^	38.18 ± 1.2^a^	32.36 ± 1.1^b^	23.56 ± 0.77^c^
Lymphocyte (%)	65.17 ± 1.13^b^	59.6 ± 1.7^ab^	53.95 ± 1.06^c^	59.4 ± 1.26^ab^	66.26 ± 0.8^a^
Monocyte(%)	4.86 ± 0.4	4.74 ± 0.4	4.64 ± 0.14	5.1 ± 0.09	6.5 ± 0.3
Esinophil (%)	3.1 ± 0.3	3.36 ± 0.3	3.09 ± 0.28	2.96 ± 0.3	3.39 ± 0.08
Basophil (%)	0.27 ± 0.03	0.17 ± 0.03	0.13 ± 0.03	0.13 ± 0.03	0.27 ± 0.03

Data represent means ± pooled SEM. Values with different letters are significantly different (*P* < 0.05). Values with the same letter are not significantly different (*P* > 0.05).

**Table 6:** Serum biochemical indices of nile tilapia, *Oreochromis niloticus* fed the experimental diets for 85 days.

Parameters	Test diets				
	R0	R10	R20	R30	R40
Total serum protein (g dL^− 1^)	2.9 ± 0.03^b^	3.3 ± 0.6^ab^	3.8 ± 0.1^a^	3.4 ± 0.05^ab^	3.1 ± 0.08^b^
Glucose (mg dL^− 1^)	11.5 ± 0.1	11.13 ± 0.4	11.3 ± 0.15	11.5 ± 0.1	11.5 ± 0.05
Triglycerides (mg dL^− 1^)	102.3 ± 1.5	103.67 ± 2.7	105 ± 3.06	105.6 ± 0.6	101.6 ± 0.6
Total cholesterol (mg dL^− 1^)	87.6 ± 0.5	81.45 ± 2.67	81.1 ± 1.1	83.1 ± 0.2	83.7 ± 0.3
Alanine amino transferase (IU/I)	2.7 ± 0.05	2.6 ± 0.23	2.55 ± 0.08	2.63 ± 0.01	2.68 ± 0.01
Aspartate aminotransferase (IU/I)	69.5 ± 1.6^ab^	66.6 ± 0.4^ab^	66.1 ± 1.5^ab^	68.8 ± 1.4^ab^	71.7 ± 0.9^a^

Data represent means ± pooled SEM. Values with different letters are significantly different (*P* < 0.05). Values with the same letter are not significantly different (*P* > 0.05).

### Intestinal histomorphological indices

**Fig. 2:**
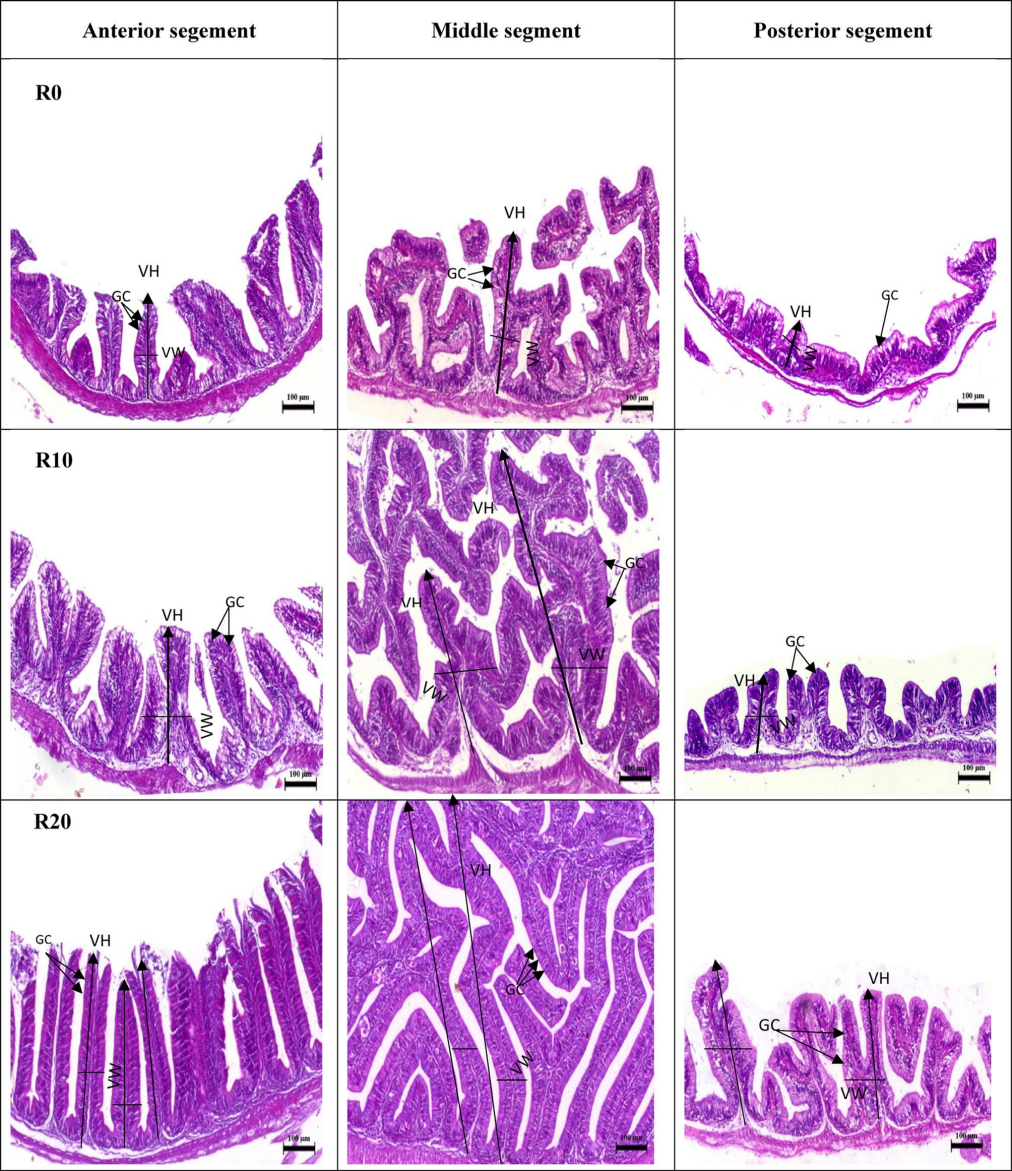

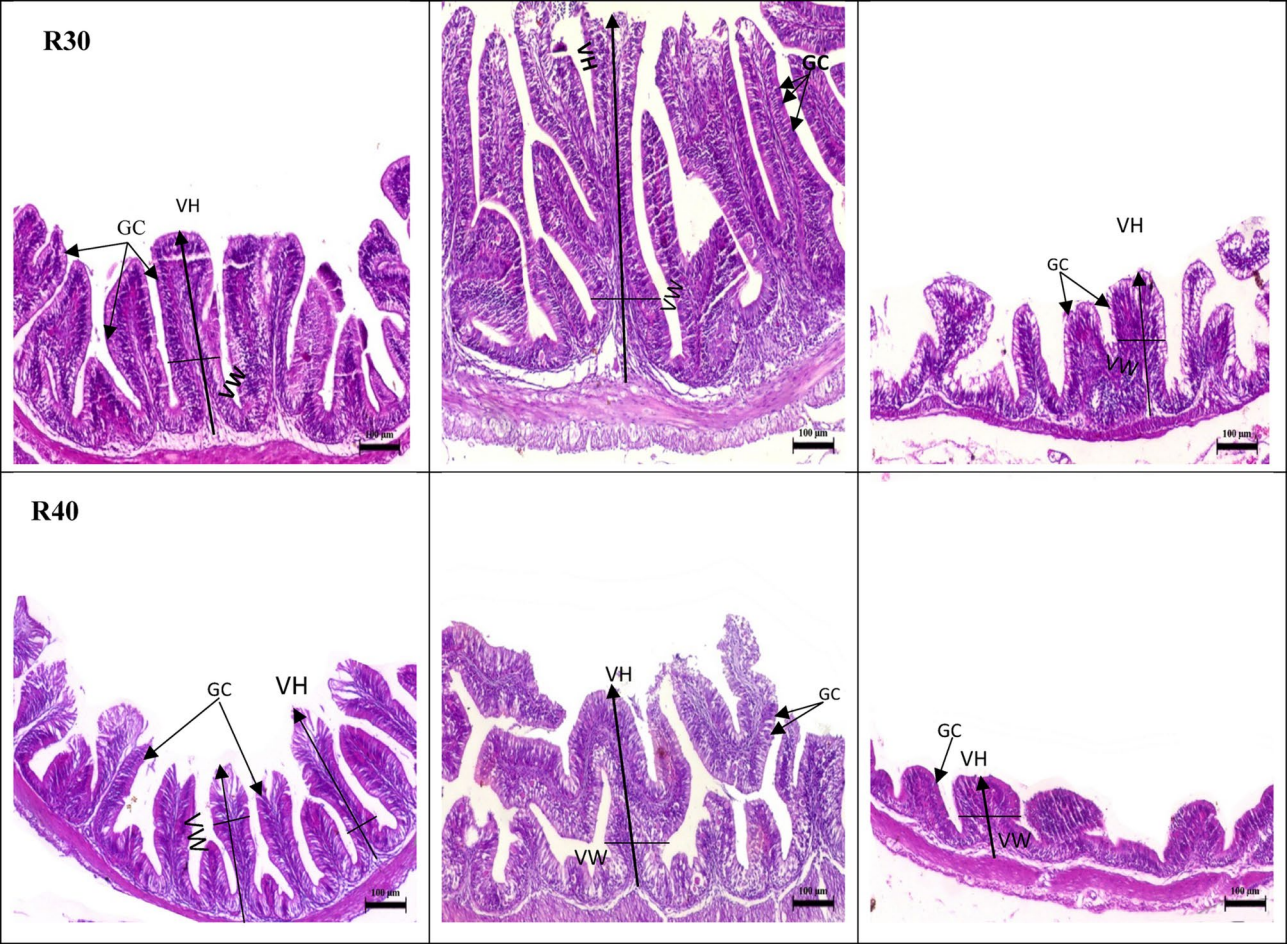
Anterior, middle, and posterior photomicrograph, bar = 100 m of the Nile tilapia’s intestine (bar = 100 μm, stain H&E). The anterior and middle parts of the control group (R0) had normal branching villi, propria submucosa, lamina muscularis, whereas the posterior section displayed normal mucosal folds. The anterior and middle parts of R10,R20 and R30 showing branching and increasing villi length, whereas the posterior section displayed normal mucosal folds.R40 tested group demonstrated normal intestinal villi length in the anterior and posterior segment, whereas the posterior segment demonstrated normal villi length, and normal intact mucosal folds. VH: villi height; VW: villi width; GC: goblet cell.

Histological examination of the intestines revealed the normal structure of the intestinal wall, mucosal fold, and intestinal villi in all segments (anterior, middle, and posterior) in the control group (R0) (Fig. 2,R0). It is noteworthy that the progressive increase in FRP concentration led to a noticeable improvement in villous height and goblet cells in the anterior, middle, and posterior segments, especially at the higher levels (R20 and R30) (Fig. 2,R20 and R30).

The villi height in the anterior segment were significantly higher (*P < 0.05*) in fish fed R20, R30, and R40 compared to fish fed R10 and R0, but there was no significant difference between fish fed R30 and R40 (*P˃0.05*) (Table 7) (Fig. 2). Conversely, the fish fed R0 had the lowest villi height (*P < 0.05*) when compared to the other groups. The goblet cells numbers were significantly higher in two groups, R20 and R30 only versus other tested and control groups R40,R10 and R0,respectively. There was no noticeable influence on the villi width of the anterior region (*P˃0.05*).

In the middle segment, Fish fed R10, R20, and R30 had significantly higher villi heights (*P < 0.05*) than fish given R40 and R0 (Table 7). The highest villi height was recorded for fish in group R20 followed by R30. Goblet cells number was higher in fish-fed R20, while the lowest was in fish-fed R40 (*P* < 0.05). In the posterior segment, the highest villi length and width demonstrated higher levels in fish-fed R20, while the lowest was in fish-fed R0 (*P* < 0.05) (Table 7) (Fig. 2). Goblet cells number was higher in fish-fed R20, while the lowest was in fish-fed R0 (*P* < 0.05).

**Table 7:** Micromorphology of the nile tilapia intestine, *Oreochromis niloticus* fed test diets for 85 days.

	Parameters	Test diets				
		R0	R10	R20	R30	R40
Anterior	VilliLength (µm)	227.07 ± 15^b^	247.01 ± 8.4^b^	332.54 ± 26.9^a^	296.9 ± 13.7^ab^	258.2 ± 12^ab^
	VilliWidth (µm)	103.4 ± 18.03	118.4 ± 28.9	127.8 ± 7.1	115.7 ± 11	99.02 ± 2.8
	gobletcells	23.67 ± 2.3^b^	30.67 ± 1.2^b^	53.3 ± 5.9^a^	47.67 ± 1.9^a^	25.67 ± 1.45^b^
Middle	VilliLength (µm)	372.25 ± 22.3^d^	541.55 ± 36.8^c^	835.47 ± 17.3^a^	673.48 ± 32.6^b^	317.7 ± 20.7^d^
	VilliWidth (µm)	72.45 ± 2.5	93.87 ± 13.4	99.5 ± 11.1	82.9 ± 3.2	88.35 ± 9.86
	gobletcells	56.3 ± 3.5^cd^	75 ± 5.6^bc^	117 ± 6.2^a^	80 ± 2.3^b^	42.3 ± 2.2^d^
Posterior	VilliLength (µm)	92.1 ± 11.4^d^	146.4 ± 16^bc^	261.2 ± 10.3^a^	170.3 ± 3.3^b^	115.8 ± 4.8^cd^
	VilliWidth (µm)	94.47 ± 40.1^b^	110.5 ± 14.3^ab^	115.6 ± 5.8^a^	111.5 ± 16.4^ab^	102.4 ± 18.8^b^
	gobletcells	12.3 ± 1.5^c^	21 ± 2.1^b^	42.67 ± 1.8^a^	27 ± 1.5^b^	20.67 ± 0.67^b^

Data represent means ± pooled SEM. Values with different letters are significantly different (*P* < 0.05). Values with the same letter are not significantly different (*P* > 0.05).

### Digestive enzyme activity

The lipase, amylase and protease activity were markedly higher (*P* < 0.05) in fish-fed R10, R20,and R30 than in fish-fed R40 and R0 (Table 8). Additionally, fish fed R20 showed highest lipase, amylase and protease activity than fish fed other diets (*P* < 0.05)(Table 8).

**Table 8:** The digestive enzyme activity of nile tilapia fed fermented and non-fermented rice polishings.

Parameters	Test diets				
	R0	R10	R20	R30	R40
Lipase activity (unit/mg)	1.42 ± 0.08^c^	1.7 ± 0.05^b^	2.4 ± 0.06^a^	1.9 ± 0.05^b^	1.16 ± 0.03^c^
Amylase activity (unit/mg)	2.7 ± 0.16^c^	3.7 ± 0.19^bc^	6.18 ± 0.4^a^	5.08 ± 0.05^b^	2.67 ± 0.14^c^
Protease activity (unit/mg	0.45 ± 0.05^c^	0.77 ± 0.04^b^	1.18 ± 0.04^a^	1.03 ± 0.09^ab^	0.39 ± 0.05^c^

Values are described as means ± SE. Different letters indicate significant differences (*P* < 0.05).

### Immune responses

**Fig. 3:**
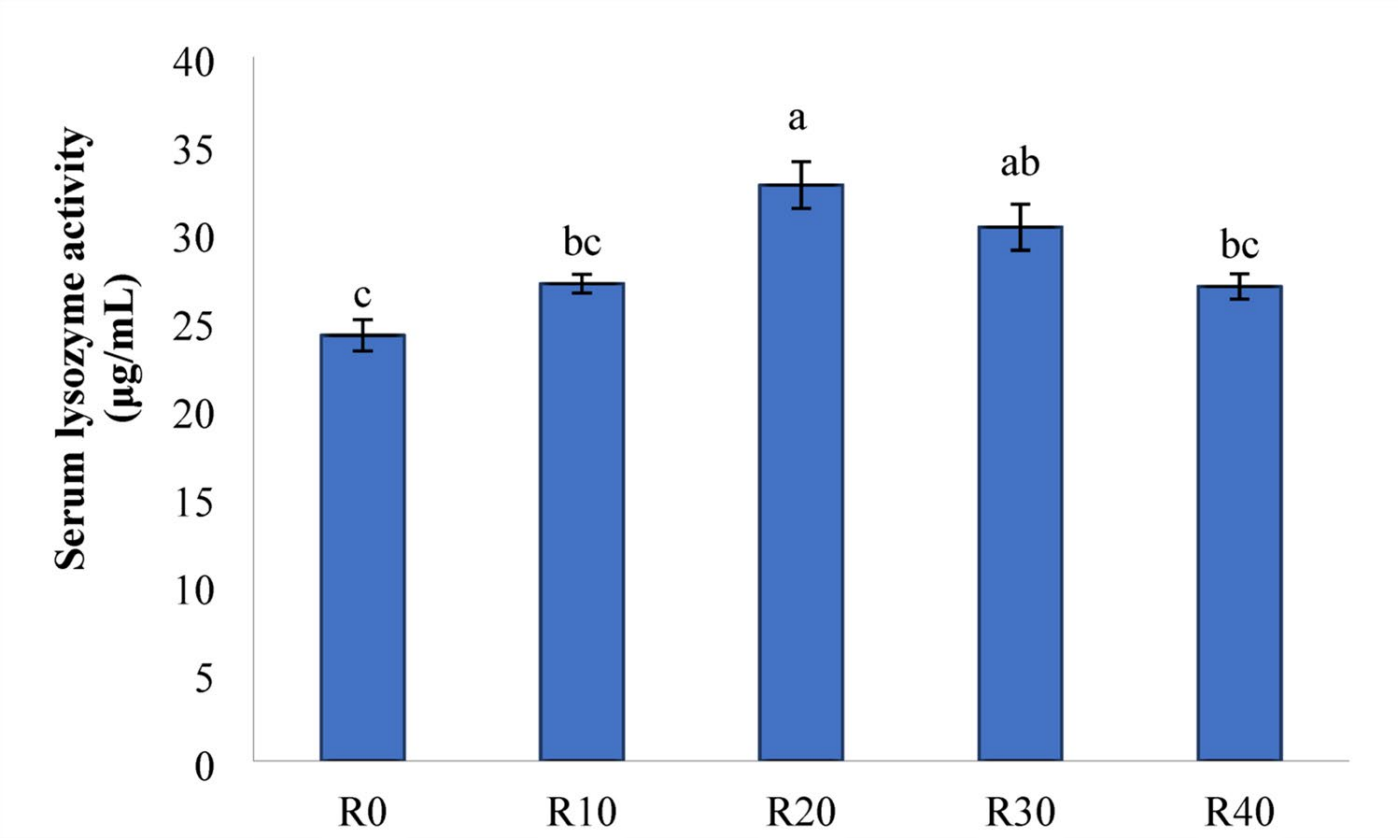
Lysozyme activity of serum (µg/mL) in Nile diet fed test diets. Data represent means ± pooled SEM. Values with different letters are significantly different (*P* < 0.05).

**Fig. 4:**
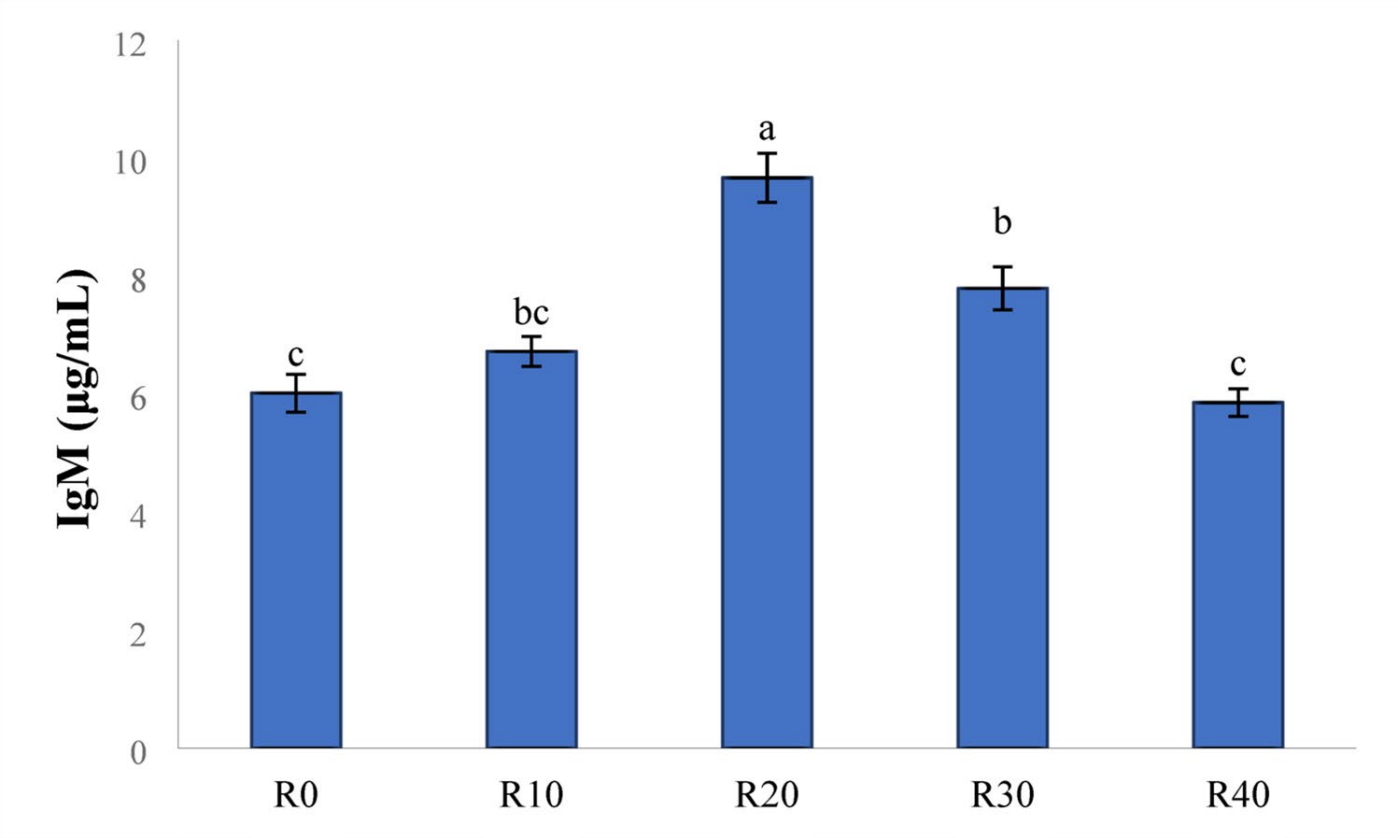
Serum Immunoglobulin M (µg/mL) level in Nile diet fed test diets. Data represent means ± pooled SEM. Values with different letters are significantly different (*P* < 0.05).

In comparison to the control group (R0), the lysozyme activity was significantly increased with all FRP-containing diets, and the highest lysozyme activity was observed in the R20 group (*P < 0.05*) (Fig. 3). IgM levels were increased significantly in fish fed FRP incorporated diets up to R30 (*P < 0.05*) (Fig. 4) compared to R40 and control group (R0). Furthermore, tilapia fed R20 had the highest IgM activities, followed by R30 and R10 respectively (*P < 0.05*).

### Antioxidant activities

**Fig. 5:**
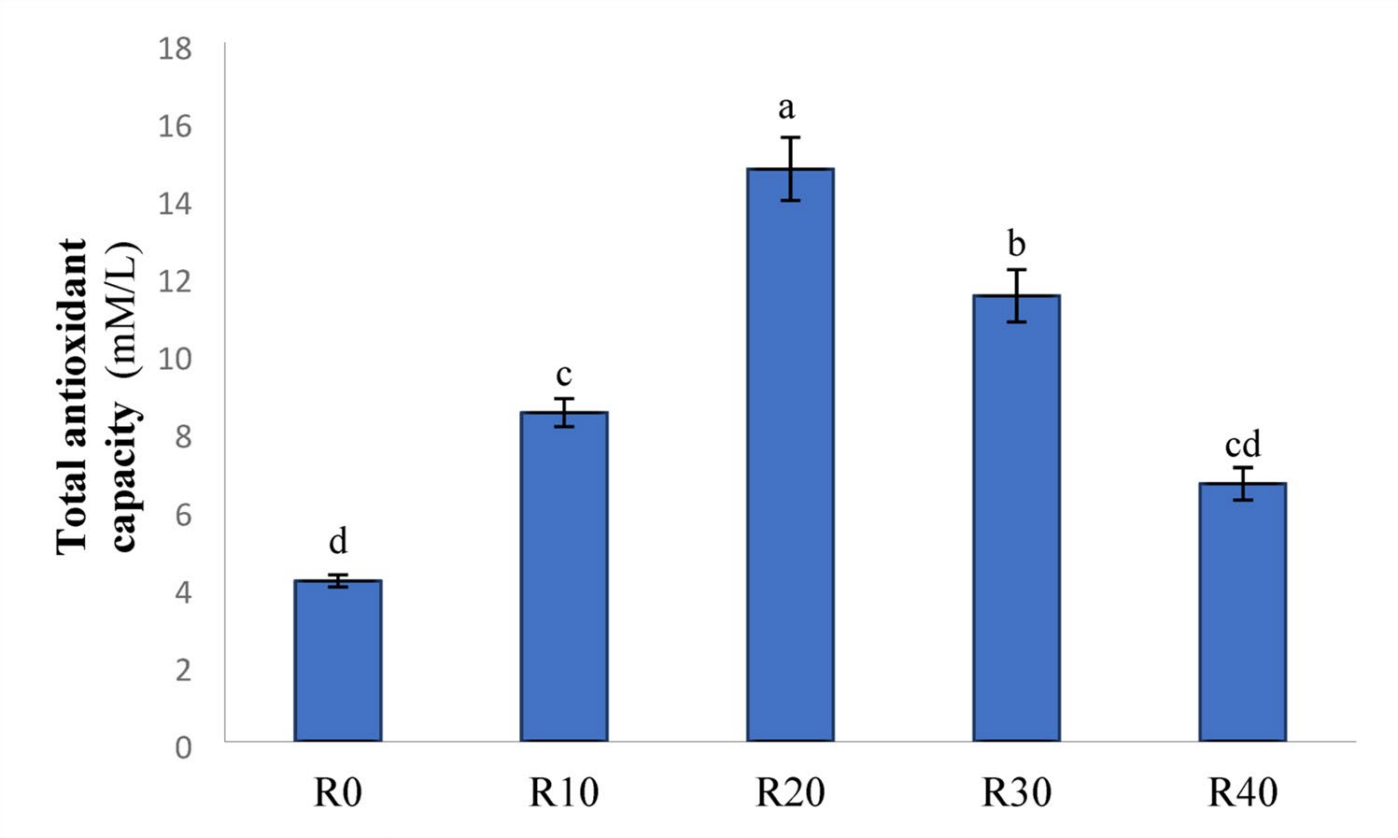
Total antioxidant capacity (mM/L) in Nile diet fed test diets. Data represent means ± pooled SEM. Values with different letters are significantly different (*P* < 0.05).

**Fig. 6:**
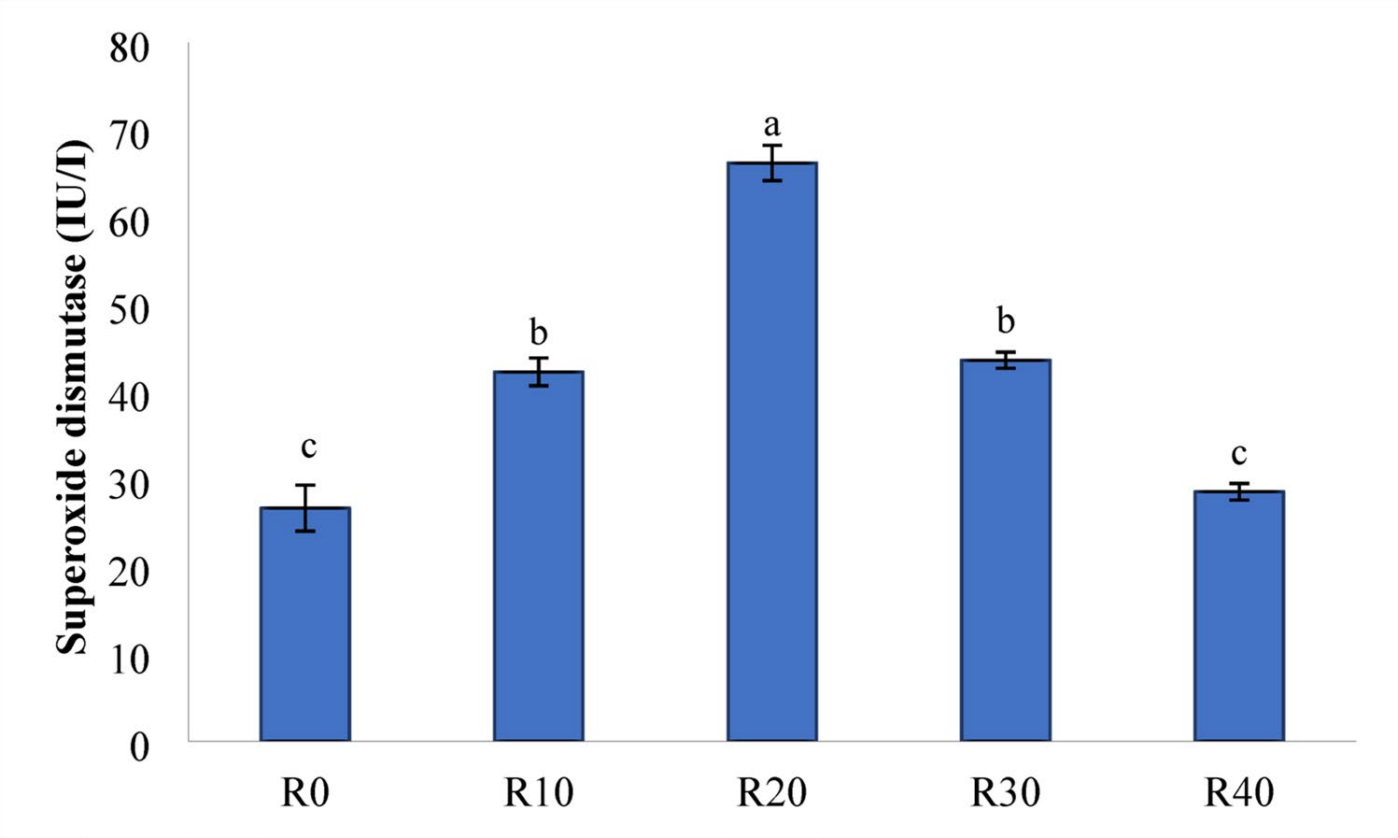
superoxide dismutase of serum (IU/I) in Nile diet fed test diets. Data represent means ± pooled SEM. Values with different letters are significantly different (*P* < 0.05).

**Fig. 7:**
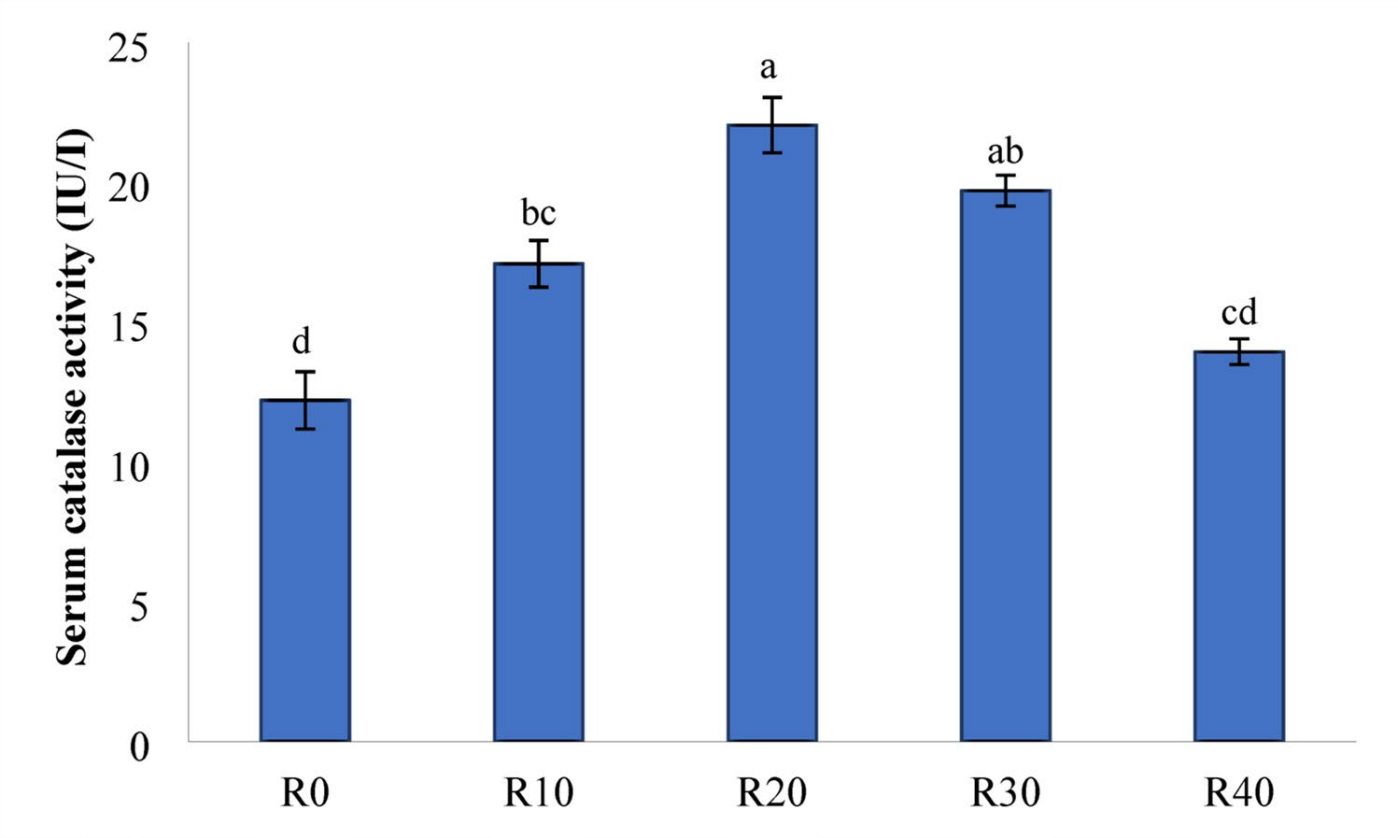
Serum catalase activity (IU/I) in Nile diet fed test diets. Data represent means ± pooled SEM. Values with different letters are significantly different (*P* < 0.05).

Figures 5 and 6, and 7 demonstrate the TAC (mM/L), SOD (IU L^− 1^), and CAT (IU L^− 1^) levels in Nile tilapia given experimental diets for 85 days. Compared to the control group (R0), FRP-containing meals significantly increased TAC and CAT activity (*P < 0.05*).While, R20 showed the greatest activity across the FRP groups (*P < 0.05*). further more, Nile tilapia given experimental diets R10, R20, and R30 had significantly higher SOD activity compared to R40 and the control group (R0) (*P < 0.05*).

### Economic evaluation

The economic evaluation of dietary FRP in the diets of Nile tilapia is shown in Table 9. The cost of feed needed for 1 kg fish fed R0, R10,R20,R30 or R40 at 0, 100, 200, 300, and 400 g/kg is 0.62, 0.56, 0.53, 0.55, and 0.54 USD/kg weight gain, respectively. The relative cost of feed per kg fish gain showed 6, 7.2, 8.66, and 10.75% reduction rates in fish-fed R10,R20,R30 or R40 at 100, 200, 300, and 400 g FRP/kg compared to fish-fed R0-based diet.

**Table 9:** Economic assessment of fermented rice Polishings incorporation in the diets of nile tilapia.

Parameters	Test diets				
	R0	R10	R20	R30	R40
Feed cost (USD/kg)	0.464	0.436	0.431	0.424	0.414
Feed conversion ratio	1.33	1.28	1.24	1.29	1.31
Cost of feed for 1 kg fish (USD/kg weight gain)	0.62	0.56	0.53	0.55	0.54
Cost reduction of feed per 1 kg fish (USD/kg weight gain)	0	0.028	0.033	0.04	0.05
Relative cost reduction of feed per kg fish gain (%)	0	6	7.2	8.66	10.75

## Discussion

Rice polishings are regarded as one of the most important byproducts of brown rice milling, due to their high nutritious content and large annual production^15^. However, because to its poor processing qualities, high fiber contents, unpleasant taste, and ease of rancidity, it is not completely utilized in the animal feed^15^. Consequently, fermentation technologies have been effectively used to improve the digestibility and solubility of fiber-rich byproducts^35^. In recent years, there has been more research on the total processing and utilization of rice byproducts to increase its utilization rate in addition to the thorough utilization of nutritional fiber, protein, and oil^36–38^.

Fermentation biotechnology have been effectively employed to increase the solubility and digestibility of byproducts that are high in fibers. In this study, we investigated the incorporation of fermented rice polishings (FRP) in the diet of Nile tilapia to enhance the digestibility of rice polishings that contain high fiber content. The findings showed that FRP could be added to Nile tilapia diet without affecting the fish’s ability to develop, their gut health, or their immune and antioxidant levels. The graded levels of fermented RP provided to Nile tilapia resulted in improved growth performance outcomes, including final weight, weight increase, and SGR. It’s notable to note that employing yeast to ferment RP resulted in decreased phytic acid content (6.24 + 0.09 to 1.23 + 0.03 mg g-1), and crude fiber content (19.2 ± 0.17 to 9.5 ± 0.23%), while increased protein levels (12.67 ± 0.2 to 17.25 ± 0.56%). This result may be attributed to the ability of *S. cerevisiae* to hydrolyze and convert fibers into proteins. As well as, Yeast may use carbohydrates as a source of carbon required for the synthesis of proteins^39–41^. In a similar way employing yeast fermentation to wheat bran^42^, sunflower meal^43^, soybean meal^44^, and rapeseed meal^45^ resulted in higher protein levels and reduced fiber contents. The improved growth performance outcomes could also be attributed to the beneficial effects of feed fermentation, which improved the fiber breakdown of the RP and encouraged efficient feed utilization and increased body weight^46–48^. Similarly, the fermentation of wheat bran by *S. cerevisiae* improved the growth performance of Nile tilapia^42^. The positive functions of S. cerevisiae as a fermenter and probiotics, according to the authors, are linked to the enhanced growth performance of Nile tilapia fed yeast fermented wheat bran and date palm seed meal^49–51^. The findings are consistent with those of ^52,53^, who found that feeding Nile tilapia fermented guar and copra meal and wheat protein concentrate, as well as *S. cerevisiae*, increased feed efficiency.

The superior growth performance observed at 200 g/kg fermented rice polishing (RP) inclusion compared to 400 g/kg can be explained by several factors. At the optimal 200 g/kg level, RP provided a balanced supply of essential amino acids and energy that synergized effectively with other dietary components to support protein synthesis and growth^54,55^. In contrast, the higher 400 g/kg inclusion exceeded Nile tilapia’s capacity to tolerate antinutritional factors, particularly phytic acid, whose concentration increased proportionally with FRP levels and significantly impaired nutrient digestibility^56,57^. Furthermore, the elevated dietary fiber content at 400 g/kg FRP likely reduced both feed utilization efficiency and gut transit function, creating additional metabolic constraints that collectively limited growth performance^58,59^.

Fermentation has several benefits, one of which is the elimination of anti-nutritional factors (ANFs), which improves intestine digestion, absorption, and nutrient release^60,61^. These factors will illustrate the growth performance enhancement and enhanced feed utilization (FCR and PER) obtained after inclusion of the graded levels of FRP in the diet of tested Nile tilapia in this study. Since the intestines are the organs in responsible for digesting and absorbing feed, being healthy might also indicate efficient feed utilization, research has shown that fermented feeds may regulate the intestinal bacteria load, promoting intestinal health and effective digestion^62^. Moreover, Fish development and performance are intimately linked to the activity of digestive enzymes, and the obtained findings showed that dietary FRP enhanced the activity of the digestive enzymes (lipase, amylase, and protease). Likewise, *S. cerevisiae*-fed Nile tilapia fermented date palm seed meal resulted in improved digestive enzyme activity^49,50^. In addition to their functions in fermentation, *S. cerevisiae* is a well-known probiotic that may release digestive enzymes throughout fish the gastrointestinal tract, promoting efficient utilization of nutrients and digestion, which in turn promotes efficient metabolic process^63,64^. Since the intestines are the organs in responsible for digesting and absorbing feed, being healthy might also indicate efficient feed utilization.

According to ^65^, hematological indices are regarded as a useful and trustworthy indicator of fish wellbeing as well as physiological and pathological conditions. According to previous research, the blood parameters measured in this study are considered to be within the typical ranges for Nile tilapia. Additionally, feeding on the experimental diets did not substantially alter the level of WBC, heterophil, triglycerides, cholesterol, ALT, lymphocytes, monocytes, eosinophils, or basophils, suggesting that FRP added to the diet of Nile tilapia was completely safe. The ability of blood to carry and transport dissolved oxygen is mostly dependent on hemoglobin, hematocrit, and red blood cells^50^. Values for these metrics were substantially higher in the FRP groups, suggesting that the fish’s health had improved. This result is consistent with earlier research on aquatic species, both wild and cultivated, which showed that high hematocrit, hemoglobin, and red blood cell counts were linked to rapid movement and high activity^66^.

While there was largely no significant difference in ALT activity across the experimental groups, significantly decreased levels of AST were recorded when Nile tilapia were fed a diet containing FRP, particularly R20 and R30. This is an indication that feeding tilapia FRP-supplemented diets could influence the liver health of fish positively^67^.

Insoluble fibers, ANFs, and silica in RP may affect the histological characteristics of fish intestines^68,69^. The effects on the intestinal epithelial tissue also depend on the quantity of ingredients, the length of consumption, and the animal species^70^. The outcomes demonstrated that the control group had normal intestinal mucosa and intestinal wall structure throughout all segments (anterior, middle, and posterior). Additionally, the integration of FRP induced a significant improvement in villus height in the anterior section and enhanced branching of intestinal villi (villus height and breadth) in the middle and posterior segments, especially at the higher levels (RP20 and RP30). These results were in harmony with^42,49,53^, who observed that *S. cerevisiae* fermented wheat bran, date palm seed meal, and wheat protein concentrate improved the intestinal histological measures in Nile tilapia, fermented rapeseed meal in red sea bream^48^. When fish were fed graded amounts of FRP, the villi length in the middle segment increased, which was linked to an increase in goblet cell counts. These benefits support the concept that enhanced digestion and absorption capacity are directly connected to improved villi surface^71^. The improved intestinal morphology—characterized by increased villi height, greater goblet cell numbers, and enhanced digestive enzyme activity—observed with moderate fermented rice polishing (FRP) inclusion (200–300 g/kg) likely results from two key mechanisms. First, fermentation generates bioactive compounds (short-chain fatty acids, peptides, and prebiotics) that directly stimulate epithelial cell proliferation (promoting villi elongation), upregulate mucus production (increasing goblet cell counts), and enhance enzyme secretion through cellular signaling^72,73^. Second, the fermentation process reduces anti-nutritional factors like phytic acid, thereby improving mineral bioavailability for gut barrier maintenance while simultaneously decreasing inflammatory responses that could compromise mucosal integrity^74^. In contrast, the diminished effects at 400 g/kg FRP may be due to the fiber overload that could accelerate intestinal transit (reducing nutrient absorption time) and alter microbial production; and the physiological capacity to process FRP-derived nutrients may be exceeded beyond optimal inclusion levels, reflecting a threshold effect common in nutritional studies^58^.

Fermentation with beneficial bacteria generates short-chain fatty acids (SCFAs), which are easily transported across the epithelium and enhance the permeability of absorbed nutrients^75^. Furthermore, fermentation reduces the adverse consequences of ANFs on epithelial function. This ultimately results in efficient feed utilization, which contributes to superior growth performance and fish health^76^. As a result, efficient feed utilization (FCR and PER) was reported in this study, which can explain improved growth performance with dietary FRP. Feed utilization and digestion have a strong influence on the chemical makeup of fish carcasses^77^. Since certain digested nutrients can accumulate throughout the fish’s entire body, this demonstrates effective feed utilization. Consequently, the investigation found no obvious impacts of FRP on the protein, ash, and moisture contents of Nile tilapia carcasses. However, FRP, particularly the FRP 20, R30, and R40 diets, had a noticeable impact on the lipid content. Because of their functions in stimulating development and aiding in digestion, dietary *S. cerevisiae* may have an impact on the composition of fish carcasses^50^. This can also be related to the high lipid content in the tested rice polishings, which may affect the composition of the fish carcass^78^.

One of the most reliable blood parameter tools for assessing the immunological and physiological status of fish is total serum protein, which may be influenced by the quality of animal feed^79,80^. Similar to this, natural antibodies (IgM) have a variety of defence functions, including preventing the spread of infectious agents, eliminating pathogens, healing damaged tissue, and re-establishing a healthy status^81^. In the current study, the immunological condition (total serum protein and total IgM) of Nile tilapia was considerably improved by the graded levels of FRP, particularly a diet enriched with the R20. All FRP groups showed a significant rise in IgM levels, according to our data, with R20-supplemented fish showing the greatest values. These findings are in line with earlier research that shown the contribution of *saccharomyces* and fermentation in raising blood IgM levels^50^.

Further, lysozyme is an important innate immune system defensive mechanism that mediates protection against microbial invasion^82,83^. Lysozyme activity was enhanced when fermented rice polishings were incorporated in the Nile tilapia diet, especially at the level of R20 & R30. The explanation for this could be related to the probiotic ability of *Saccharomyces cerevisiae* to increase lysozyme secretion in Nile tilapia, and or the benefit of fermentation that counteracting the effect of antinutritional factors and high fiber contents of FRP^4,42,49^.

Malnutrition, the presence of ANFs, and insoluble fibers in RP may all cause the production of free radicals, which is mediated by antioxidant biomarkers such as TAC, CAT, and SOD^84^. CAT is an important antioxidant enzyme found in nearly all biological tissues that demand oxygen, and subsequently catalyzes the breakdown or reduction of hydrogen peroxide into water and molecular oxygen, therefore completing the detoxification process initiated by SOD^85^. In particular, CAT protects cells from oxidative damage by using radical scavenging enzymes to convert hydrogen peroxide into water^86^. SOD is an essential antioxidant enzyme that disperses two molecules of superoxide anion to produce hydrogen peroxide (H_2_O_2_) and molecular oxygen (O_2_)^87^. The fermentation process may enhance the release of polyphenols through the glycolysis process of plant-derived components. In this study, serum TAC, CAT, and SOD activity were considerably increased in Nile tilapia given FRP compared to the control, reaching the highest activity level in R20 group. The significant antioxidative functions of these polyphenols are well established in general health enhancement. Additionally, *S. cerevisiae* has a high concentration of MOS and β-glucan, which may improve the immunological and antioxidant response^88,89^. Similar findings were reported by^90^, who found that *S. cerevisiae* fed to Nile tilapia exhibited an activated antioxidant response.

The economic evaluation of dietary FRP revealed that Nile tilapia production costs were lower than the control group (R0). These findings suggest that implementing FRP might improve the sustainability of the Nile tilapia aquaculture business. Additional future studies are required to focus on adding suitable feed additives such as attractants, exogenous digestive enzymes, prebiotics, probiotics, and natural immunological and growth boosters to increase the FRP utilization in Nile tilapia diets. Ultimately, the results of this study demonstrate that dietary supplementation with fermented rice polishings can effectively enhance the growth performance, feed utilization, haematological indices, immunological parameters, antioxidant status, and digestive function of Nile tilapia. The high nutritious content present in rice polishings, derived through the fermentation process, appear to be responsible for the overall beneficial outcomes discovered throughout numerous physiological indices. The optimal dietary incorporation level was determined to be 210–230 g/kg diet, which provided the most significant improvements. These findings reveal the potential of the rice polishings as a valuable functional plant by-product to improve the productivity and health of Nile tilapia in aquaculture systems.

## Conclusion

This study demonstrates that fermented rice polishings (FRP) can be effectively incorporated into Nile tilapia diets at up to 30% without adverse effects on growth, nutrient utilization, immune response, or oxidative status, while 20% FRP emerges as the optimal inclusion level for maintaining fish health and economic viability. The findings highlight FRP’s dual value as both a sustainable feed ingredient that valorizes agro-industrial waste and a functional supplement that enhances gut health and antioxidant capacity, with 20% inclusion offering the best balance between performance and safety. Notably, the negative effects observed at 40% inclusion underscore the importance of dosage control to prevent intestinal damage and growth reduction. These results provide practical guidance for tilapia farmers seeking to reduce feed costs while maintaining fish health, contributing to more sustainable aquaculture practices through the effective utilization of rice milling byproducts.

## Supplementary Information

Below is the link to the electronic supplementary material.

Supplementary Material 1

## Data Availability

All data supporting the results of this research are accessible upon request from the corresponding author.
