# Supplementary material for: Fermented rice polishings dietary supplementation sustainably enhanced growth performance, gut morphology, immune response and antioxidant status of Nile tilapia
Source: Sci Rep. 2025 Nov 21;15:41162. doi: 10.1038/s41598-025-26417-y (PMC12639150; doi:10.1038/s41598-025-26417-y)
Supplement: Supplementary file 1 — Supplementary Material 1 [file 41598_2025_26417_MOESM1_ESM.docx]

**Table S1**

**NRC Dietary Guidelines and the Proposed Experimental Diet Plan**

| **Nutrient** | **NRC (2011) Recommendation** | **Control Diet (No FRP)** | **FRP-Supplemented Diet** | | | |
| --- | --- | --- | --- | --- | --- | --- |
|  |  |  | **R10** | **R20** | **R30** | **R40** |
| Crude Protein (%) | 28–32 | **27.4** | **27.3** | **27.44** | **27.65** | **27.5** |
| Lipid (%) | 5–8 | **4.72** | **5.13** | **5.63** | **6.63** | **7.62** |
| Fiber (%) | <8 | **3.48** | **4.59** | **5.64** | **6.68** | **7.68** |
| Energy (KJg^-1^) | 19-21 | **19.29** | **19.32** | **19.4** | **19.65** | **19.77** |

Nutrient Requirements of Fish and Shrimp, NRC 2011,(Council 2011)

Council, N. R. (2011). Nutrient Requirements of Fish and Shrimp. Washington, DC, The National Academies Press.

Aquaculture now supplies half of the seafood and fisheries products consumed worldwide and is gaining international significance as a source of food and income. Future demands for seafood and fisheries products can only be met by expanded aquaculture production. Such production will likely become more intensive and will depend increasingly on nutritious and efficient aquaculture feeds containing ingredients from sustainable sources. To meet this challenge, Nutrient Requirements of Fish and Shrimp provides a comprehensive summary of current knowledge about nutrient requirements of fish and shrimp and supporting nutritional science. This edition incorporates new material and significant updates to information in the 1993 edition. It also examines the practical aspects of feeding of fish and shrimp. Nutrient Requirements of Fish and Shrimp will be a key resource for everyone involved in aquaculture and for others responsible for the feeding and care of fish and shrimp. It will also aid scientists in developing new and improved approaches to satisfy the demands of the growing aquaculture industry.
